# 20^th^ century Betula pubescens subsp. czerepanovii tree- and forest lines in Norway

**DOI:** 10.3897/BDJ.5.e22093

**Published:** 2017-11-23

**Authors:** Anders Bryn, Kerstin Potthoff

**Affiliations:** 1 University of Oslo, Oslo, Norway; 2 Norwegian Institute of Bioeconomy Research, Ås, Norway; 3 Department of Geography, Bergen, Norway

**Keywords:** elevation, mountain birch, range limit, re-mapping, spatial coordinates, treeline

## Abstract

**Background:**

Georeferenced tree- and forest line data has a wide range of applications and are increasingly used for e.g. monitoring of climate change impacts and range shift modelling. As part of a research project, registrations of previously re-mapped tree- and forest lines have been georeferenced. The data described in this paper contains 100 re-mapped registrations of Betula pubescens subsp. czerepanovii throughout Norway. All of the re-mapped tree- and forest line localities are georeferenced, elevation and aspect are given, elevational and spatial uncertainty are provided, and the re-mapping methods are explained. The published data weremapped for the first time between 1819 and 1963. The same sites were re-mapped between 1928 and 1996, but have until now been missing spatial coordinates. The entries contain 40 x 2 tree lines and 60 x 2 forest lines, most likely presenting the regionally highest registered tree- and forest lines at the given time. The entire material is stored and available for download through the GBIF server.

**New information:**

Previously, the entries have been published in journals or reports, partly in Norwegian or German only. Without the provision of the spatial coordinates, the specific locations have been unknown. The material is now available for modelling and monitoring of tree- and forest line range shifts: The recordings are useful for interpretation of climate change impacts on tree- and forest lines, and the locations of re-mapped tree- and forest lines can be implemented in future monitoring projects. Since the recordings most likely provide the highest registered Betula pubescens subsp. czerepanovii locations within their specific regions, they are probably representing the contemporary physiognomic range limits.

## Introduction

The last century of global warming affects the world’s vegetation, particularly in cold temperature-limited ecosystems (e.g. Hudson and Henry 2009, Pauli et al. 2012). The two most striking vegetation boundaries along the elevational gradient at northern latitudes, are i) the transition from forest to low-alpine areas with scattered trees (forest line) and ii) the uppermost single trees towards treeless alpine areas (treeline). Both vegetation boundaries (termed TFLs) are primarily limited by low temperatures (Holtmeier 2009, Körner 2012), restricting establishment and height growth, and regulating survival and die-back. A general agreement exists that the TFLs of Norway are moving upwards (e.g. Rannow 2013) and northwards (e.g. Hofgaard et al. 2012). Recent reviews of TFL changes however, have been built on very few and spatially scattered entries from Norway (e.g. Cudlín et al. 2017). Previous re-sampling studies of TFLs from Norway have not been systematically reviewed the last decades, and the data availability has been restricted by the analog format. In addition, the exact locations of the previous re-sampling studies have never been georeferenced, and are partly published in Norwegian or German. To enable renewed re-sampling and to document historical TFL distributions, we have georeferenced 100 locations of re-mapped TFLs of Betula pubescens subsp. czerepanovii (N.I. Orlova) Hämet-Ahti from Norway.

## General description

### Purpose

The purpose of making re-mapped and georeferenced data of TFLs available was to enable the data's potential for spatiotemporal analyses of TFL dynamics. Specifically the goals were to identify the localities, georeference them, and through GBIF publish re-mapped TFL locations of Betula pubescens subsp. czerepanovii from Norway.

### Additional information

The data described in this article contains 100 georeferenced entries of re-mapped TFLs of Betula pubescens subsp. czerepanovii. The data contains registrations of elevation from two periods, a first registry and a second re-mapped registry from the same site. Each entry consists of re-mapped TFL localities that are georeferenced. The elevation and exposition is given for both records. Elevational and spatial uncertainty is provided for all entries, and the re-mapping methods are explained. The entries have recently been published (18 October 2017) on the GBIF-server, they are stored there, and are available for download.

## Sampling methods

### Study extent

The study extent includes mainland Norway.

### Sampling description

The TFLs have originally been re-sampled in six different ways (Table 1): i) Most of the re-sampling has been carried out by in-situ re-mapping (79 entries), that is to re-visit locations during field-work. ii) Nine entries have been re-sampled through in-situ comparison of old and young forest. The age of the young forest at higher elevation, is then contrasted with the elevation of older forest at lower altitudes. iii) Eight entries were registered through comparing empirical TFLs with climatic TFLs, i.e. documenting elevational difference between lowest land use disturbed TFLs with uppermost climatic TFLs. iv) One entry was re-sampled by comparing the present day forest line with the historical forest line identified on an old photograph, whereas v) one forest line entry was re-sampled based on oral information on the elevation of previous forest line. vi) Two entries were re-sampled by comparing previous TFLs with an updated map.

The TFL locations which have been georeferenced, were spatially located through three steps using GIS (ArcGIS v. 10.3). First, the locality names provided by the authors were used to locate sites based on a query in the standard Norwegian topographic maps (NMA 2017). If locality names had changed, they were identified with the help of contemporary analog maps (historical maps). Second, we located the provided aspect on an aspect map derived from a 20 m digital elevation model over Norway. Third, we identified the most likely position at the reported elevation, by interpretation of both old and new aerial photos.

The georeferenced locations have two main potential sources of uncertainty: i) the uncertainty given by the original authors regarding the measurements of elevation and ii) the uncertainty regarding the exact spatial position along the combination of aspect and elevation.

The uncertainties have been reported in two ways: i) The altitudinal uncertainty (expected precision) has been divided into three categorical classes: high precision (± 5 m a.s.l.) reflects standard GPS quality, intermediate precision (± 10 m a.s.l.) reflects in-situ measurements using barometer, and comparisons of empirical and climatic forest lines, whereas low precision (± 25 m a.s.l.) reflects comparisons of old and young forest lines, comparison with old photo, map comparison, or where authors have reported uncertain measurements of elevation. ii) The uncertainty regarding coordinate precision has been reported as a vector length combining aspect and elevation at the sites. This uncertainty ranges between 160 and 5200 m, with an average of 1113 m.

### Quality control

All re-sampled records have been checked with aerial photos, and 76 of the locations (76 %) have been visited during field-work from 2013 to 2017. The oldest re-sampled records are more or less impossible to validate, since they are older than all available aerial photos and precise maps, and since most trees from that period are dead and lost through decomposition.

Twenty potential records have not been registered. Ten records were average numbers, and did not represent single locations, whereas the other ten records only showed the elevational change and not the elevation at the locations.

## Geographic coverage

### Description

**Figure 1.**
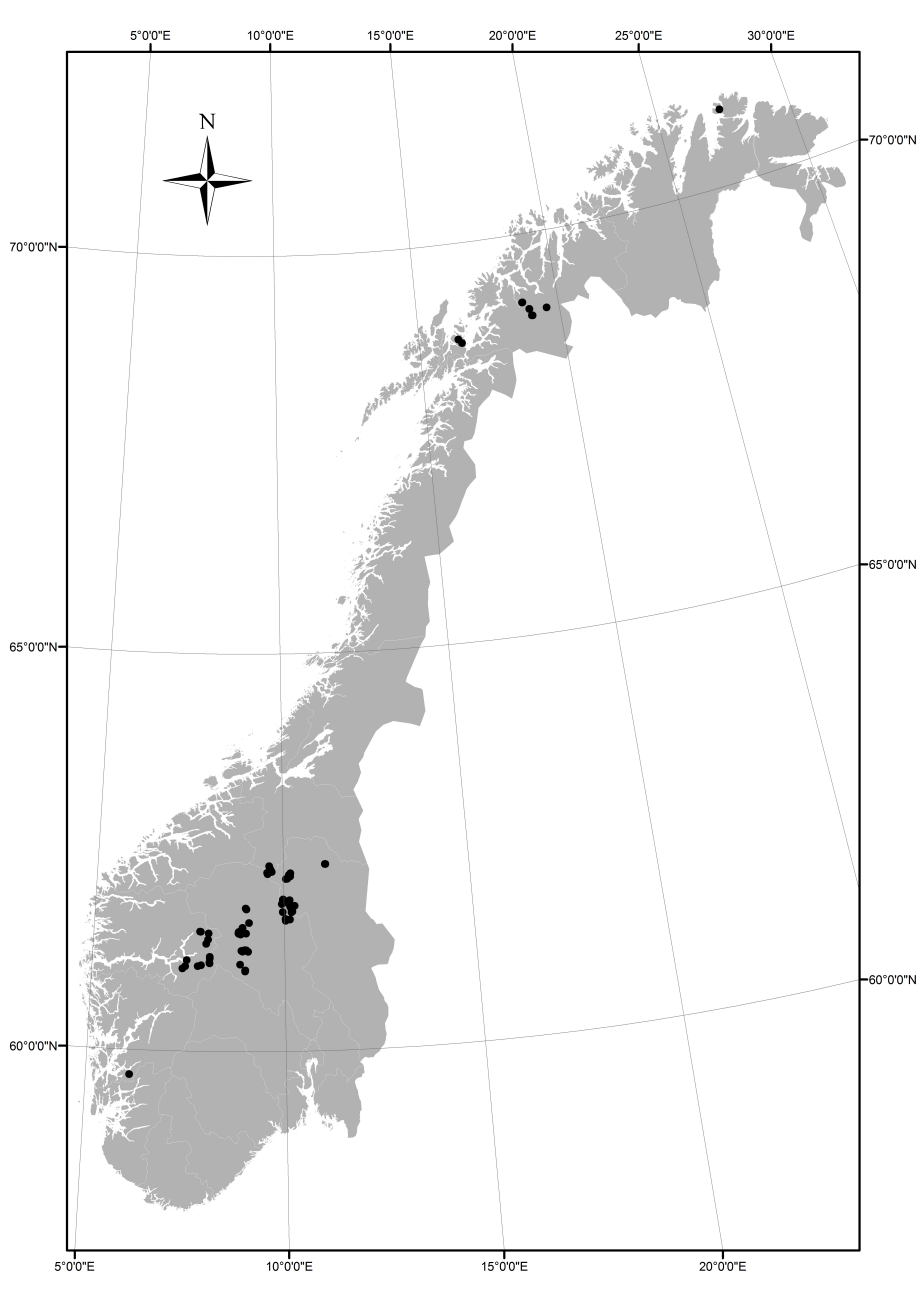
Distribution of the re-mapped tree- and forest lines in Norway.

The TFLs have been re-sampled in the main mountain regions of Norway, but some regions have far more entries than others (Fig. 1). South-central Norway has most of the entries, whereas mid Norway and southeastern Norway lack entries. The coastal mountains have few and scattered entries. The dataset has been gathered within the uppermost parts of the boreal (forest line) and the lower parts of the alpine (treeline) bioclimatic regions (Bakkestuen et al. 2008).

### Coordinates

57°58'50'' and 71°11'8'' Latitude; 4°49'8'' and 31°3'26'' Longitude.

## Taxonomic coverage

### Description

This dataset includes occurrence data from 1 species, mountain birch (Betula pubescens subsp. czerepanovii). There are two common synonyms, namely Betula pubescens subsp. tortuosa (Ledeb.) Nyman, and Betula pubescens var. pumila (L.) Govaerts. Misidentification of mountain birch (Betula pubescens subsp. czerepanovii), at the highest elevated locations, is highly unlikely. This is because it is the only known tree-forming birch species present at the boreal-alpine ecotone in Norway. In GBIF, the higher nomenclature was added to all records, from Kingdom to Subspecies.

## Traits coverage

The entries include mountain birches tall enough to be defined as trees following the definitions of the original authors (Table 1). It is thus the location of a physiognomic plant unit that has been georeferenced.

## Temporal coverage

### Notes

**Figure 2.**
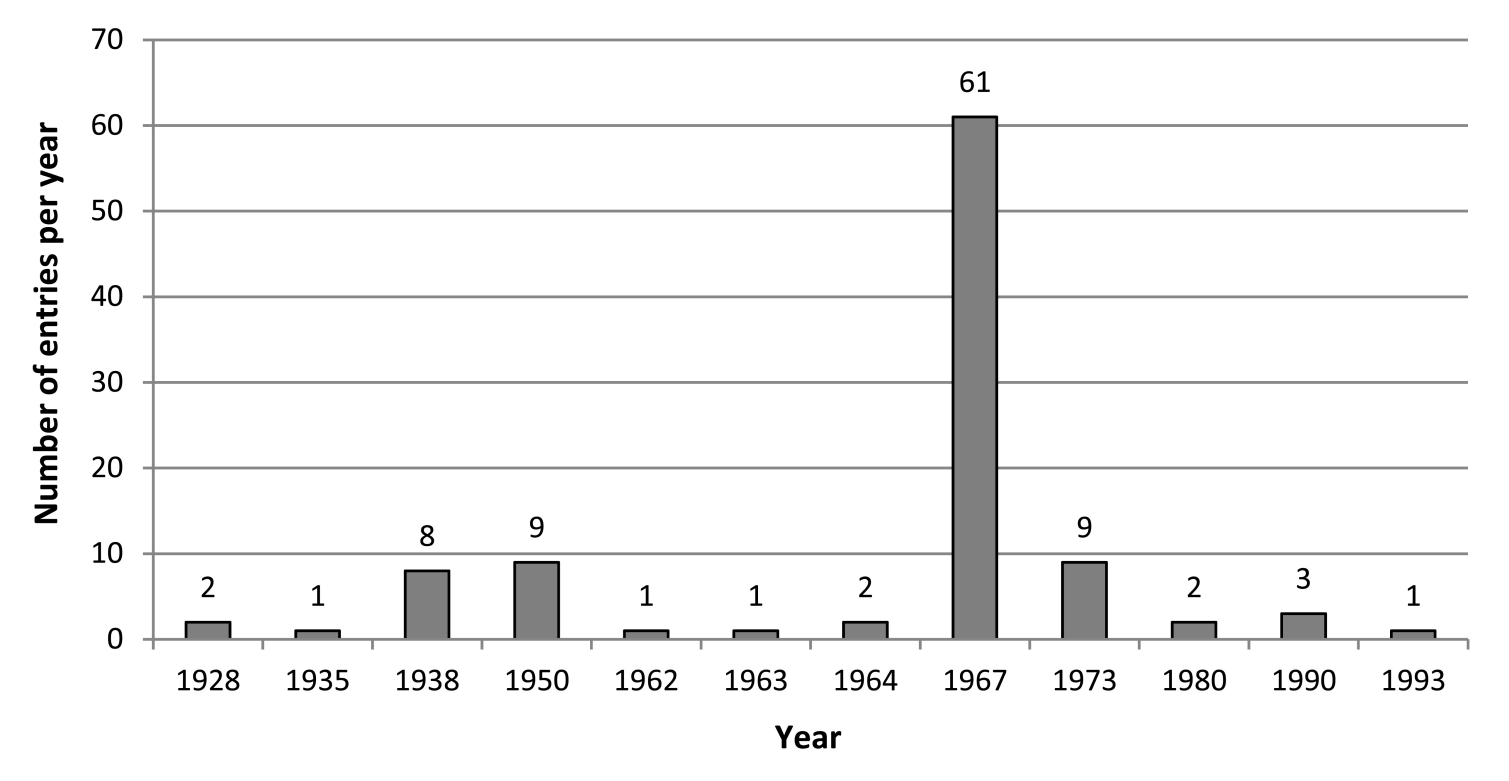
Number of re-mapped records per year.

The data havebeen re-sampled from 1928 to 1996, varying in number from 1 entry in the years 1935, 1962, 1963 and 1993 to 61 entries in 1967 (Fig. 2). The re-sampled period also varies, from 27 to 109 years. For nine entries, the year of first registry is unknown, since the method was based on comparing previously land use influenced forest lines at low elevation with contemporary high elevation forest lines (Ve 1940).

## Usage rights

### Use license

Creative Commons Public Domain Waiver (CC-Zero)

## Data resources

### Data package title

20th century Betula pubescens ssp. czerepanovii tree- and forest lines in Norway

### Resource link

http://DOI.org/10.15468/rb4gcc

### Alternative identifiers

https://www.gbif.org/dataset/ec961283-84ec-46e4-80ba-2cedb9ee51d3

### Number of data sets

1

### Data set 1.

#### Data set name

20th century Betula pubescens ssp. czerepanovii tree- and forest lines in Norway

#### Data format

Darwin Core Archive Format

#### Number of columns

41

#### Description

Occurences of re-sampled tree- and forest lines in Norway. The dataset consists of Darwin Core Attributes.

**Data set 1.** 

Column label	Column description
occurrenceID	An identifier for the occurrence
eventID	An identifier for the record (record code)
parentEventID	An identifier for the pairs of re-sampled occurrences (grouping of events)
organismID	An identifier for the organism instance
dcterms:modified	The most recent date-time on which the resource was changed
institutionCode	The acronym of the institution providing the record
datasetName	The name identifying the data set from which the record was derived
scientificName	The full scientific species name, including authorship information
basisOfrecord	The specific nature of the data record
kingdom	The full scientific name of the kingdom in which the taxon is classified
phylum	The full scientific name of the phylum in which the taxon is classified
class	The full scientific name of the class in which the taxon is classified
order	The full scientific name of the order in which the taxon is classified
family	The full scientific name of the family in which the taxon is classified
genus	The full scientific name of the genus in which the taxon is classified
specificEpithet	The name of the species epithet of the scientificName
infraspecificEpithet	The name of the terminal infraspecific epithet of the scientificName, excluding any rank designation
scientificNameAuthorship	The authorship information for the scientificName formatted according to the conventions of the applicable nomenclaturalCode
recordedBy	Names of the person(s) who has recorded data
year	The four-digit year in which the Event occurred, according to the Common Era Calendar
month	The ordinal month in which the Event occurred
country	The name of the country in which the location occurs
stateProvince	The name of the county in which the data werecollected
locality	The specific name of the locality
verbatimLongitude	The verbatim original longitude of the location
verbatimLatitude	The verbatim original latitude of the location
verbatimCoordinateSystem	The spatial coordinate system for the verbatimLatitude and verbatimLongitude
decimalLongitude	The geographic longitude (in decimal degrees, using the spatial reference system given in geodeticDatum) of the geographic center of a location
decimalLatitude	The geographic longitude (in decimal degrees, using the spatial reference system given in geodeticDatum) of the geographic center of a location
geodeticDatum	The ellipsoid, geodetic datum, or spatial reference system (SRS) upon which the geographic coordinates given in decimalLatitude and decimalLongitude as based
coordinateUncertaintyInMeters	The coordinate uncertainty of the specific locality given in meters
georeferencedBy	Names of those who determined the georeference (spatial representation) for the location
verbatimElevation	The original description of the elevation (altitude above sea level) of the location, in meters
minimumElevationInMeters	The lower limit of the range of elevation (altitude above sea level) of the location, in meters
maximumElevationInMeters	The higher limit of the range of elevation (altitude above sea level) of the location, in meters
associatedReferences	Reference to the original literature associated with the occurrence
dynamicProperties	Aspect of the locality given as categories (E, SE, S, SW, W, NW, N, NE)
eventRemarks	Provides a record of the two physiognomic units that were measured at the location; tree line or forest line
samplingProtocol	A short name describing the method for re-sampling tree- and forest lines
eventRemarks	Specific comment regarding uncertainty or method
samplingProtocol	A short name describing the type of physiognomic range limit (single highest or lowest)

## Figures and Tables

**Table 1.** Reference to original author and number of entries of tree- and forest lines.

**Original author (reference)**	**Tree lines**	**Forest lines**	**Re-sampling method**
Aas and Faarlund 1995	0	3	In-situ re-mapping (1), photo comparison (1) and map comparison (1)
Aas and Faarlund 1996	0	5	In-situ re-mapping (4) and map comparison (1)
Aas 1969	36	25	In-situ re-mapping
Axelsen 1975	2	7	In-situ re-mapping
Ekrheim 1935	0	1	Oral information
Skar 1964	0	2	In-situ re-mapping
Ve 1930	0	2	In-situ re-mapping
Ve 1940	0	8	Comparison of empiric and climatic forest
Ve 1951	2	7	Comparison of old and young forests
Total	40	60	
